# Unplanned adnexectomy for ovarian cystadenoma with undiagnosed autoamputation of the contralateral ovary, lessons learned from medical mistakes

**DOI:** 10.52054/FVVO.13.2.017

**Published:** 2021-06-28

**Authors:** A Daccache, E Feghali, R Assi, Z Sleiman

**Affiliations:** Lebanese American University Medical Center – Rizk Hospital.

**Keywords:** Mature cystic teratoma, torsion, autoamputation, medical errors, complications

## Abstract

Autoamputation of the ovary is a rare occurrence of uncertain aetiology with only a few cases reported in literature. It usually develops following ovarian torsion or torsion of a dermoid cyst with subsequent necrosis of the pedicle and autoamputation. We present the case of a 42 year-old woman was admitted for a laparoscopic removal of a right ovarian cyst. The ultrasound showed a right ovarian cystic mass suggestive of a cystadenoma, and another heterogeneous small echogenic cyst of the left ovary. During laparoscopy, excessive bleeding from the ovarian cortex complicated the cyst stripping and, considering the age of the patient and the emerging technical difficulty of the procedure, a total adnexectomy for the right ovary was performed. While exploring the small cyst on the left ovary, a dermoid cyst was found in the Douglas pouch. This finding could be interpreted as an autoamputation of the adnexa due to an asymptomatic torsion of a previous ovarian cyst arising from the left ovary. Medical errors could occur due to lack of knowledge, expertise, as well as lack of training and surgical skills, but also due to an unfortunate association of very rare confounding factors. Even in the hands of experts, following the basic rules of surgery remains a milestone in teaching and preventing surgical complications.

## Introduction

Surgical practice is a dynamic specialty and a fertile field for potential errors and complications. Such incidents could occur with any type of surgery despite full adherence to strict protocols and in the hands of trainees as well as skilled expert surgeons. In the specific setting of the laparoscopic approach, a higher risk of major complications was observed in abdominal surgeries and/or the presence of technical difficulties related to the procedure itself. Another important cause of complications utilising the laparoscopic route can be due to the training process and the learning abilities of the surgeon. Indeed, hours of dry lab training are required to acquire the basic psychomotor skills before stepping up in the operation theatre (Sleiman et al., 2015).

As such, the gravity of an error can be major or minor, but in few cases a major complication may occur because of a simple medical mistake. We herein report a case of auto amputation of the ovary, an exceptional scenario that led to the commitment of a major medical error.

## Case presentation

**Figure 1:**
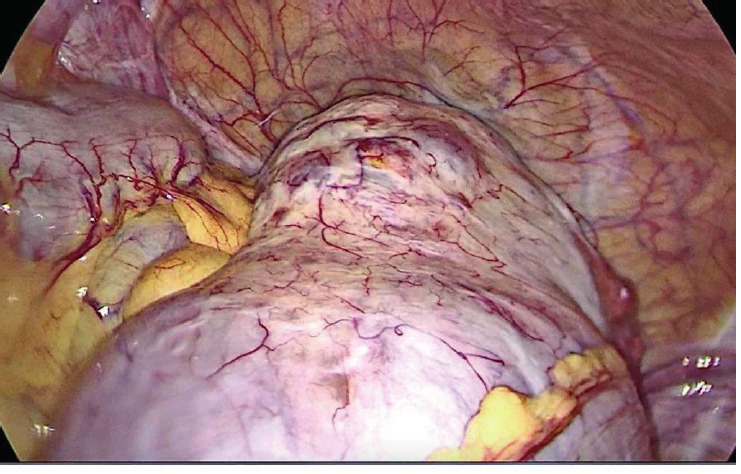
— The left ovarian mass obliteration of the pelvic cavity.

A 42 year-old Caucasian G3P3A0 woman was admitted to our facility for a laparoscopic removal of an 8 cm right ovarian cyst. The patient had no history of previous surgery. The ultrasound confirmed a right ovarian cystic mass suggestive of a cystadenoma, and another heterogeneous small echogenic 2 cm cyst of the left ovary. As our institution is an academic university hospital, the chief resident initiated the laparoscopic procedure under the supervision of the attending surgeon. Upon entry, the pelvis was obliterated by the pelvic mass of the ovary (Figure 1).

**Figure 2:**
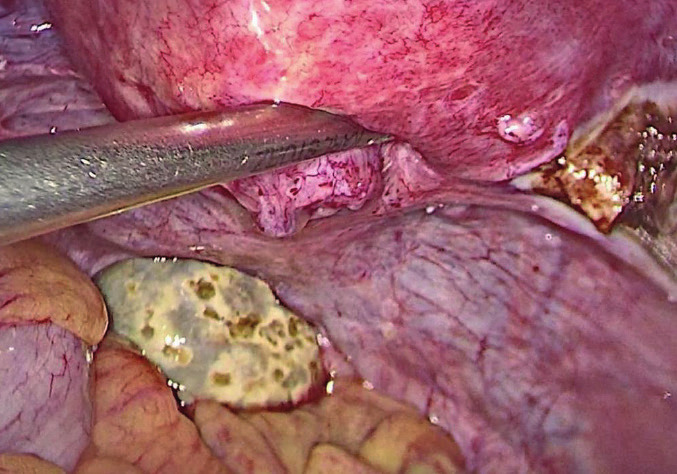
— The difficult dissection of the cystic wall.

**Figure 3:**
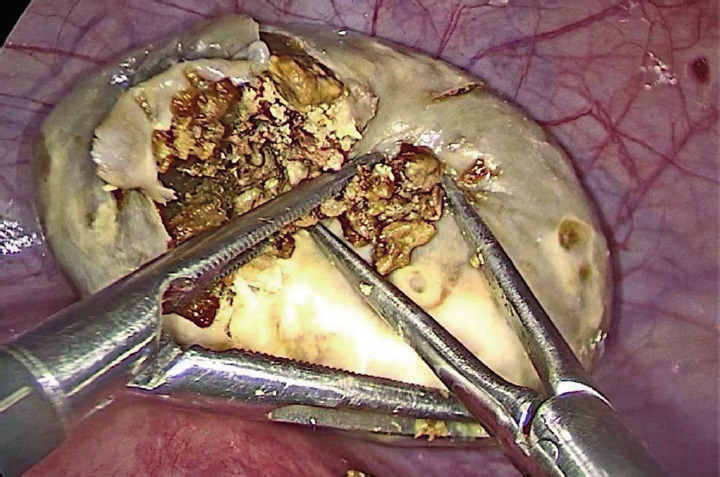
— The floating autoamputated left ovary in the pouch of Douglas.

**Figure 4:**
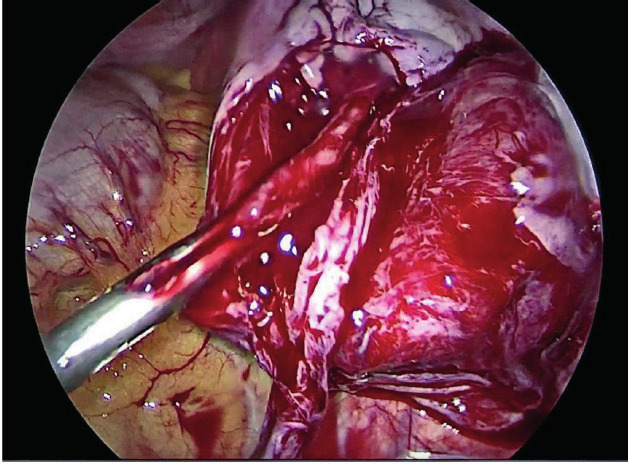
— The fatty content of the amputated left ovary.

The resident physician started by cutting the ovarian cortex and dissecting the cystic wall. Excessive bleeding from the ovarian cortex complicated the cyst stripping and therefore it was not an easy step (Figure 2). We then decided to perform a total adnexectomy for the right ovary, considering the age of the patient and the emerging technical difficulty of the overall procedure. We subsequently proceeded to explore the small cyst of the left ovary. To our surprise, we could not find it at the fossa ovarica; instead, the ovary was totally detached and found in the Douglas pouch (Figure 3). When we dissected the detached mass, the content was consistent with a dermoid cyst (Figure 4). Both adnexa were then removed using an endobag, and the tissue type of both cysts was confirmed with histopathology examination.

## Discussion

### 

Autoamputation of the ovary is a rare occurrence of uncertain etiology with only a few cases reported in literature. It usually develops following ovarian torsion or torsion of a dermoid cyst with subsequent necrosis of the pedicle and autoamputation. The detached ovary can remain free-floating in the peritoneal cavity at different locations, or it can develop its own collateral circulation and re-implant as a parasitic ovary. The greater omentum is the usual site of implantation as it potentially plays a role in intraabdominal inflammation and formation of adhesions, thereby facilitating the secondary implantation of the autoamputated ovary (Kim et al., 2017).

A dermoid cyst can also develop in a supernumerary ovary or in a congenital ectopic ovary (Shah et al., 2019). Lee et al. (2016) reported a case of a parasitic ovarian teratoma located in the sub-hepatic space with simultaneous torsion of the left ovary. Only 12 cases of teratomas were reported in the pouch of Douglas with 6 of these thought to be due to auto amputation as the teratomas contained traces of ovarian tissues (Ohshima et al., 2015).

Furthermore, two cases of auto amputation were reported as incidental findings during caesarean section. Peitsidou et al. (2009) reported a case of an 8x5cm teratoma incidentally found in the Douglas pouch and attached with a vascular pedicle to the omentum and intestine. The patient reported a history of acute abdominal pain during adolescence that is most likely explained by torsion. Gupta et al. (2015) reported another case of an intraperitoneal but free-floating auto amputated ovary also incidentally found at the time of a caesarean section.

While the majority of ovarian torsion cases present with acute abdomen, some remain asymptomatic until infarction, necrosis and subsequent auto amputation occur.

Available data suggest that an auto-amputated ovary may possibly undergo malignant transformation after implantation into the intestine or omentum, and should therefore be removed (Kusaka and Mikuni, 2007; Zampieri et al., 2009).

To the best of our knowledge, this is the first report of an association of a large cystadenoma with an asymptomatic auto amputation of the contralateral ovary due to a dermoid cyst, leading to a free- floating ovary located in the pouch of Douglas.

### Learning from the medical error

Complications during surgery do happen and are sometimes unavoidable. It is important to have a suitable knowledge of the anatomy, the physiology and the pathogenesis of the disease in addition to the technical skills in order to minimise the number and severity of complications during a surgery. When these occur, complication management is critical and determines the clinical outcome of the patient and is crucial in preventing medico-legal consequences (ESGE Special Interest Group ‘Quality, Safety and Legal Aspects’ Working Group, 2020).

In case of bleeding, the initial and fastest step to stop the bleed is by applying pressure using a clamp or any other instrument. Any attempt of blind coagulation should be avoided. Next, a decision should be made to whether or not to convert to laparotomy. This conversion decision should be made within seconds, requiring the surgeon and the resident to maintain self-control and act rapidly. For this reason, it is important to always mention to the patient pre-operatively the risk of conversion to laparotomy. Also, regular simulations are of important value in training the team on such events. (Rochlen et al., 2019). This scenario could be also preventable if the resident would have access to the cyst from the opposite side, where the plan of dissection with the ovarian cortex is clearer.

In our case, a number of confounding factors led the surgical team to commit an error by inducing menopause for the patient through adnexectomy before checking the contralateral ovary. Available data suggests that most of the medical errors are actually the result of faulty reasoning instead of lack of knowledge. The generation of hypotheses mostly relies on pattern recognition based on resemblances between cases previously encountered and the case at hand (Mamede et al., 2014). These observations largely apply to our case. In the presence of two separate ovarian cysts, we normally remove the big cyst that is obliterating the general view then we proceed to remove the smaller one. Nevertheless, the surgeon should, a priori, confirm the presence of two ovaries on ultrasound and during surgery before attempting any surgical act that could have unforeseen long-term sequelae. What made the situation more complicated is the rarity of the presented case. Indeed, what are the odds of having an asymptomatic floated auto amputated ovary hidden in the Douglas pouch in a patient with a big contralateral cystadenoma?

In the Harvard Medical Practice study (Brennan et al., 1991), patients were unintentionally harmed by treatment in around 4% of admissions. This resulted in slight or temporary disability in 70% of affected patients, permanent defect in 7%, while 14% of patients died partly because of their treatment. According to multiple studies, about half of the adverse events and errors were judged preventable (Vincent et al, 2001).

Surgical competency is a continuum involving multi-level good decision making abilities, coordination of team performance and communication, as well as technical skills. When coupled with long-term expertise and a high patient volume operating rate, this competency potentially reduces patient mortality and morbidity (Birkmeyer et al, 2002).

Committing errors in a supervised surgical environment is academically beneficial to trainees, as they can learn and understand better how to prevent such events occurring in the future. A crucial point to consider at any timepoint during training and with practice is the severity of the implications of a medical error. Creating a menopause at the age of 42 years is not a mild error, but definitely portends worse consequences if the same happened in a younger woman seeking fertility. According to Bamford et al. (2018), Surgical Boot Camps offer a suitable opportunity to develop practical skills while boosting a trainee’s self-confidence and experience. As a result, patient care may be enhanced with the a potential reduction in the rate and severity of errors. Sleiman et al. (2019) also published about the transferability of skills from the training lab to the operation theatre in order to reduce complications. Nevertheless, this could not have prevented the complication in our case, because the error was also not due to lack of surgical skills. Strong evidence suggests that non- surgical skills such as, but not limited to situational awareness, decision making, communication, teamwork and leadership affect the occurrence of unfavorable surgical events. In fact, non-technical errors are the second leading cause of operative errors (Rao et al., 2016). Decision making can also be improved by attending specific courses that incorporate practical case scenarios as part of their training to enhance the team performance.

The decision to remove the ovary involved by the cystadenoma before checking the second remaining ovary was an anonymous decision of our team. The responsible attending approved the removal of the ovary reasoning that it is impossible to safely remove the cyst. The mistake was therefore not a personal error but rather a more systematic mistake (Soane et al., 2014). Successful bodies have a specific reporting system that allocates personal responsibility without attributing blame. Because physicians are expected to be perfect, this may result in a tendency towards covering up errors instead of admitting them (Shepherd et al., 2019). Nevertheless, we admitted our error and explained to the patient all the circumstances that led to the unfortunate removal of both ovaries. Even in the hands of experts, mistakes can occur, and these mistakes can be due to the association of rare scenarios and by omitting the basic rules of surgery. An expert is most of the time called out for complicated cases and when facing surgical difficulties needing special expertise. The association of multiple factors led to a major mistake that could have been simply prevented by a simple systematic check of the anatomy.

## Conclusion

Medical error can occur due to lack of knowledge, expertise, as well as lack of training and surgical skills, but also due to an unfortunate association of very rare confounding factors. Even in the hands of experts, following the basic rules of surgery remains a milestone in teaching and preventing surgical complications.
